# *Astragalus membranaceus* Inhibits Peritoneal Fibrosis via Monocyte Chemoattractant Protein (MCP)-1 and the Transforming Growth Factor-β1 (TGF-β1) Pathway in Rats Submitted to Peritoneal Dialysis

**DOI:** 10.3390/ijms150712959

**Published:** 2014-07-22

**Authors:** Zhenghong Li, Lu Zhang, Weiming He, Changle Zhu, Jinsong Yang, Meixiao Sheng

**Affiliations:** 1 Department of Nephrology, Jiangsu Province Hospital of Traditional Chinese Medicine, Baixia Hanzhong Road 155, Nanjing 210029, China; E-Mails: njzylzh@163.com (Z.L.); alexzhanglu@aliyun.com (L.Z.); 13851699906@163.com (W.H.); 15951952026@163.com (J.Y.); 2 Department of Pathology, Jiangsu Province Hospital of Traditional Chinese Medicine, Baixia Hanzhong Road 155, Nanjing 210029, China; E-Mail: zhuchangle2008@163.com

**Keywords:** *Astragalus membranaceus*, monocyte chemoattractant protein, transforming growth factor-β1, peritoneal fibrosis, peritoneal dialysis

## Abstract

Inflammation and transforming growth factor-β1 (TGF-β1) contribute to the development of peritoneal fibrosis (PF), which is associated with peritoneal dialysis (PD). *Astragalus membranaceus* (*Astragalus*) has anti-inflammatory and anti-fibrotic effects in many diseases. The goal of this study was to determine the anti-fibrotic effects of *Astragalus* on the PF response to PD. A rat model of PD was induced using standard PD fluid, and PF was verified by HE and Masson’s staining, as well as through the expression of fibroblast surface protein (FSP) and collagen III. The expression levels of monocyte chemoattractant protein (MCP)-1, F4/80 (macrophage/monocyte marker in rat), TGF-β1 and the downstream proteins phospho-SMAD 2/3 in dialyzed peritoneal tissue treated with or without *Astragalus* was evaluated using immunohistochemistry analysis. Overall correlations between MCP-1 and TGF-β1 staining were analyzed using both the Spearman and Pearson methods. The results showed that *Astragalus* could inhibit the recruitment and activation of monocytes/macrophages, thereby reducing the production of TGF-β1 in the dialyzed peritoneal membrane. PF was also significantly decreased following treatment with *Astragalus*. MCP-1 expression had a strong positive correlation with TGF-β1 sensitivity, suggesting that the anti-fibrotic function of *Astragalus* was mediated by MCP-1 and the TGF-β1 pathway. Our results indicate that *Astragalus* could be a useful agent against PD-induced PF.

## 1. Introduction

Peritoneal dialysis (PD) and hemodialysis are well-established treatments for patients with end-stage renal disease. PD has several advantages compared to hemodialysis, including a simpler and less invasive procedure and the retention of residual renal function [1,2]. However, PD is associated with complications that can cause encapsulating peritoneal sclerosis (EPS), a serious disease with a high mortality rate [3,4] that can lead to ultrafiltration failure and eventually to the discontinuation of therapy. Thus, the percentage of PD patients is only approximately 10%–15% of the total dialysis population [5].

It is believed that the epithelial-mesenchymal transition (EMT) in mesothelial cells and, subsequently, peritoneal fibrosis, is a frequent complication of peritoneal dialysis following repeated low-grade inflammatory and pro-fibrotic insults [6,7,8], and this process is essential for the development of EPS [9,10,11,12,13,14]. Moreover, transforming growth factor-β1 (TGF-β1) is well known as a key pro-fibrotic cytokine [15] and a key mediator of the experimental and human peritoneal fibrosis associated with PD [16]. Targeting TGF-β1 and inflammation may be a good therapeutic strategy for controlling peritoneal fibrosis and EPS.

*Astragalus* (*Astragalus membranaceus*) has been used in Traditional Chinese Medicine (TCM) for thousands of years. *Astragalus* is used to protect and support the immune system, prevent colds and upper respiratory infections, lower blood pressure, treat heart disease and diabetes, and protect the liver with only a few side effects. Recently, several reports have demonstrated the anti-fibrotic effects of *Astragalus* on pulmonary fibrosis [17], liver fibrosis [18], and nephropathy [19] through the inhibition of the activation of the TGF-β1 signaling pathway. Furthermore, the potential anti-inflammatory effects of *Astragalus* have been shown in various *in vitro* studies [20,21,22,23]. For example, *Astragalus* may ameliorate chronic inflammatory skin diseases due to its anti-inflammatory activities via regulation of the intracellular ROS production, NF-κB, JAK/STAT and PI3/Akt signaling cascades as well as immune responses [24].

Our previous study also indicated that the treatment of high dose *Astragalus* could promote water clearance of peritoneum and increase the dialysis hyperfiltration in PD rats [25]. We hypothesized that *Astragalus* can reduce peritoneal fibrosis through the inhibition of the TGF-β1 signaling pathway and the inflammatory response. For this purpose, we developed a rat model to investigate whether *Astragalus* has a therapeutic effect on the PD-induced peritoneal fibrosis.

## 2. Results and Discussion

### 2.1. Effect of Astragalus on Peritoneal Fibrosis in PD Rats

#### 2.1.1. Morphological Changes

**Figure 1:**
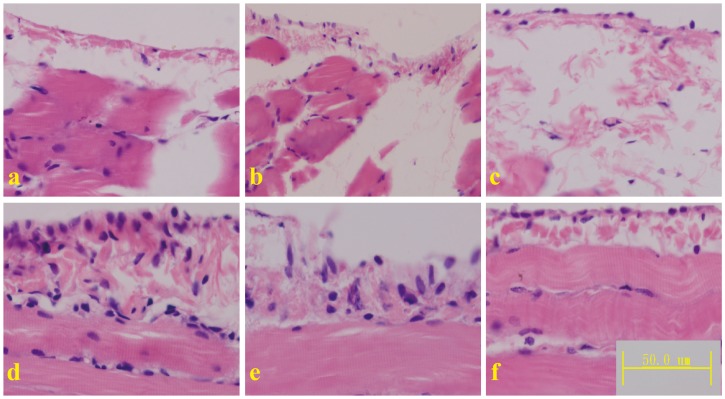
Micrographs of mesothelial cells of the diaphragmatic peritoneum from the normal control (**a**); *Astragalus* alone (**b**); peritoneal dialysis (**c**); *Astragalus* low dose (**d**); *Astragalus* middle dose (**e**); and *Astragalus* high dose (**f**) groups.

HE staining showed extensive interstitial fibrosis, mesothelial denudation and increased thickness of the submesothelial cell layer in the peritoneum of the PD rats. Compared to the PD rats, the rats treated with the middle and high doses of *Astragalus* exhibited significantly ameliorated fibrosis and thickness of the submesothelial cell layer in the peritoneum. However, *Astragalus* treatment alone did not affect the morphology of the peritoneum (Figure 1). *Astragalus* did not affect mesothelial denudation, which may have occurred as a result of damage during the sectioning process [26].

#### 2.1.2. Peritoneum Thickness

**Figure 2:**
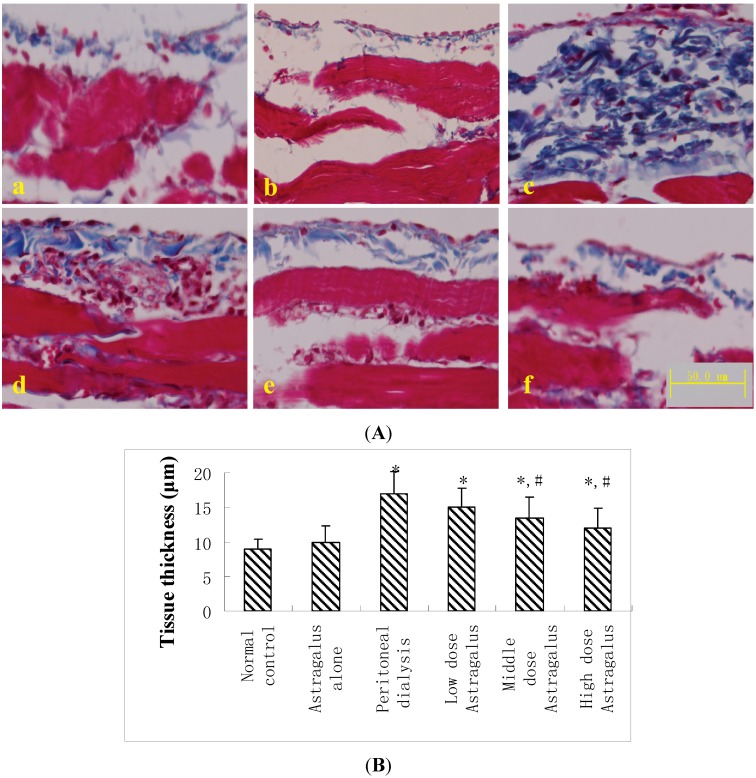
(**A**) Micrograph of Masson’s trichrome staining of the diaphragmatic peritoneum of the normal control (**a**); *Astragalus* alone (**b**); peritoneal dialysis (**c**); *Astragalus* low dose (**d**); *Astragalus* middle dose (**e**); and *Astragalus* high dose groups (**f**); (**B**) The submesothelial tissue thickness was measured in the diaphragm. Mean ± SEM. * *p* < 0.05 compared to the normal controls; # *p* < 0.05 compared to peritoneal dialysis.

According to the data from the Masson’s trichrome staining analysis, the peritoneum thickness was dramatically increased in the PD rats compared to the control and *Astragalus* alone animals. The restoration effect of *Astragalus* on the increased peritoneum thickness in the PD rats was observed in the middle and high dose groups (Figure 2A,B).

#### 2.1.3. Fibroblast Surface Protein (FSP) and Collagen III Expression

**Figure 3:**
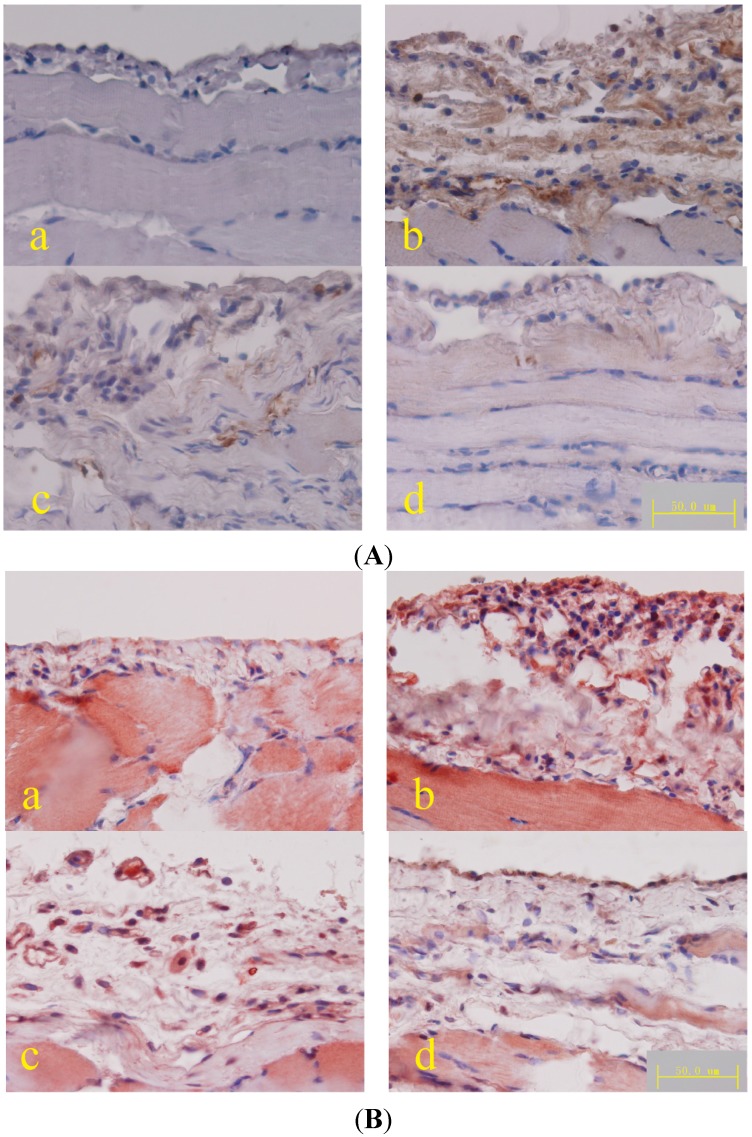
(**A**) Micrograph of FSP immunohistochemical staining of the diaphragmatic peritoneum, DAB was used as a chromogen; (**B**) Micrograph of collagen III immunohistochemical staining of the diaphragmatic peritoneum, AEC was used as a chromogen. (**a**) Normal control; (**b**) peritoneal dialysis; (**c**) *Astragalus* low dose; and (**d**) *Astragalus* high dose groups.

FSP and collagen expression in the peritoneum were increased in the PD rats but decreased in the PD rats treated with a high dose of *Astragalus* (Figure 3).

Some studies have demonstrated that peritoneal fibrosis and the subsequent EPS is always a result of long-term PD and is dependent on the duration of the PD treatment. In this study, after treatment with PD solution for 4 weeks, several pathological alterations in the peritoneum were observed in the PD rats, including extensive interstitial fibrosis, mesothelial denudation, and changes in the peritoneum thickness, as previously described in the literature [26,27]. Immunohistochemical staining revealed extensive deposition of extracellular matrices, including type III collagen and fibroblast recruitment (increased FSP-positive staining) [28,29]. These results indicated that long-term treatment with the PD solution did result in peritoneal fibrosis, and this animal model is suitable to test the effect of *Astragalus*. In the present study, *Astragalus* was shown to effectively improve peritoneal fibrosis in a dose-dependent manner. For instance, the middle and high dose of *Astragalus* significantly ameliorated the pathological alterations in the peritoneum, such as the interstitial fibrosis and the thickening of the peritoneum. Furthermore, *Astragalus* also exhibited inhibitory effects on fibroblast recruitment and collagen synthesis in the PD peritoneum. These results suggested that *Astragalus* exhibited an anti-fibrotic effect on peritoneal fibrosis, in agreement with results described in other tissues [17,18,19]. 

Based on these results, we chose the high dose of *Astragalus* for subsequent experiments to determine the underlying mechanisms of these effects.

### 2.2. Effect of Astragalus on MCP-1 Expression and Macrophage Recruitment in PD Rats

Monocytes and macrophages, whose flow from the bloodstream is mediated by chemokines secreted from resident cells, are the principle cells found at sites of inflammation. Monocyte chemoattractant protein (MCP)-1 is the most effective chemokine, and it plays an important role in the pathogenesis of various inflammatory and fibrotic diseases, including diabetic nephropathy [30], renal fibrosis [31], and cardiac fibrosis [32]. MCP-1 has also been suggested to have a functional role in the initiation and progression of PF via the recruitment and activation of monocytes/macrophages [33].

**Figure 4:**
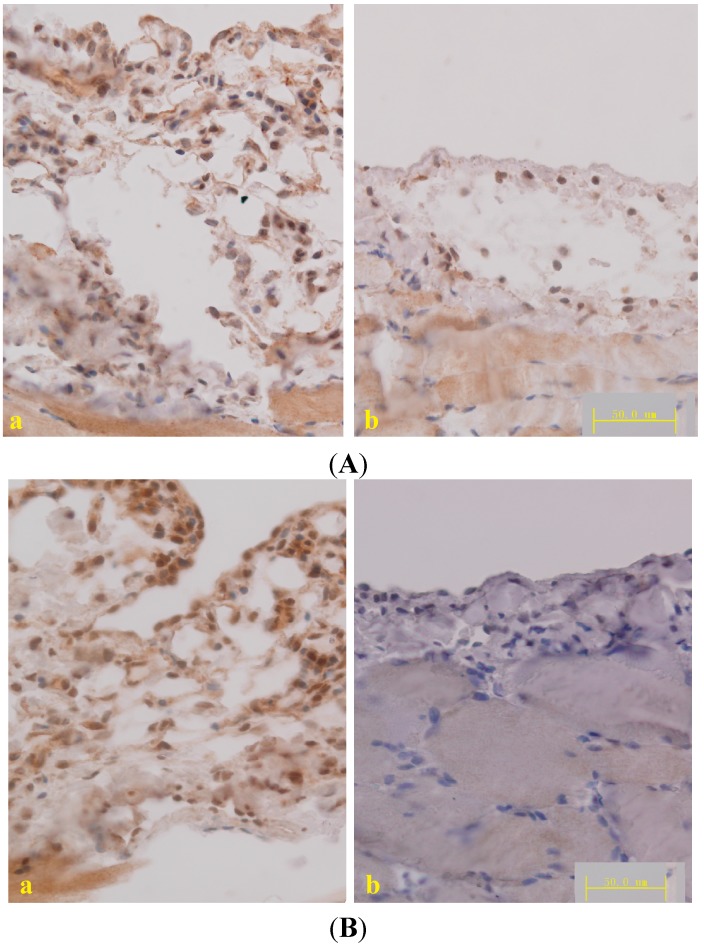
(**A**) Micrograph of MCP-1 immunohistochemical staining in the diaphragmatic peritoneum; (**B**) Micrograph of F4/80 immunohistochemical staining in the diaphragmatic peritoneum. (**a**) Peritoneal dialysis and (**b**) *Astragalus* high dose groups; DAB was used as a chromogen.

**Figure 5:**
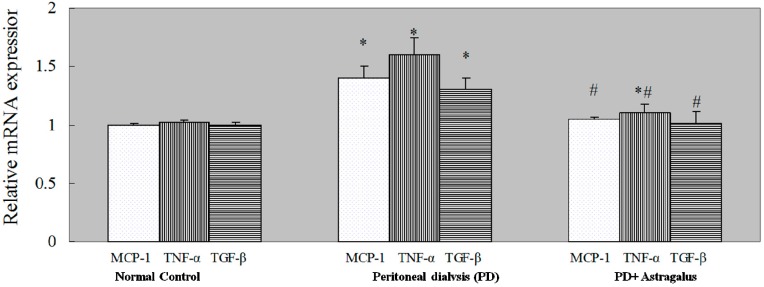
The expression of inflammatory cytokines was measured in the diaphragmatic peritoneum by qPCR. Mean ± SEM. * *p* < 0.05 compared to the normal controls; # *p* < 0.05 compared to peritoneal dialysis.

Compared to the PD rats, the number of MCP-1 positive cells was significantly decreased in the submesothelial cell layer of the peritoneum from rats treated with a high dose of *Astragalus* (Figure 4A). These results were confirmed by qPCR (Figure 5) and ELISA (Table 1). As the result of MCP-1 production, the increased number of anti-F4/80 positive cells (macrophage recruitment) in the PD rats was also reduced in the rats treated with a high dose of *Astragalus* (Figure 4B). These results indicated that *Astragalus* effectively reduced MCP-1 expression in the PD peritoneum and macrophage recruitment in the PD rats.

### 2.3. Effect of Astragalus on TGF-β1 Expression in PD Rats

**Figure 6:**
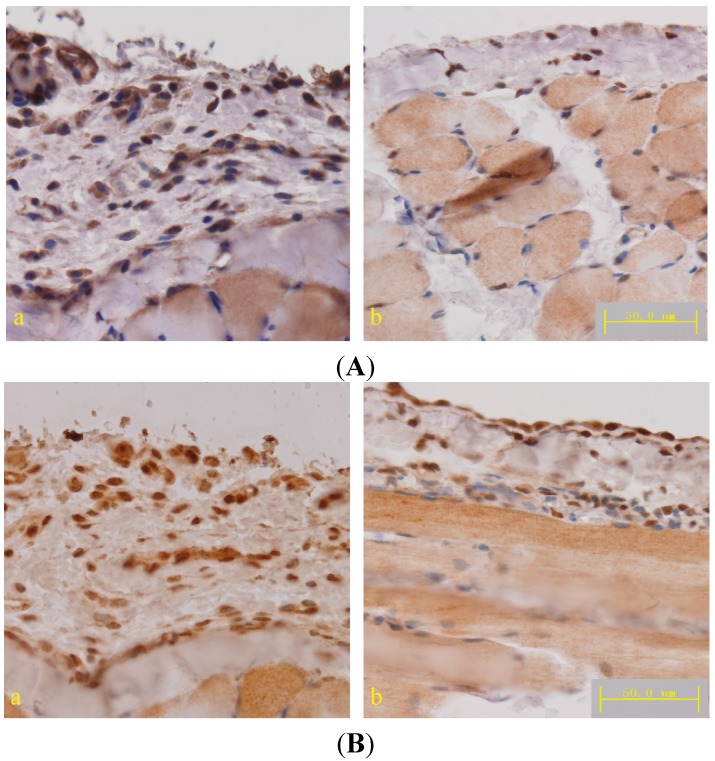
(**A**) Micrograph of TGF-β1 immunohistochemical staining in the diaphragmatic peritoneum; (**B**) Micrograph of phosphorylated Smad2 and Smad3 immunohistochemical staining in the diaphragmatic peritoneum. (**a**) Peritoneal dialysis and (**b**) *Astragalus* high dose groups; DAB was used as a chromogen.

The TGF-β/Smad pathway plays a key role in the development of fibrosis. Previous studies have demonstrated that *Astragalus* inhibits liver fibrosis [34], keloids [35], pulmonary fibrosis [17], and nephropathy [19] by mediating the TGF-β/Smad pathway. To study the mechanisms of *Astragalus* action, we tested the expression of TNF-α, the cytokine produced by macrophages, which activates TGF-β1 promoter activity [36]. The expression of TGF-β1 was determined by qPCR, ELISA and immunohistochemical staining. The downstream proteins of TGF-β1, phosphorylated Smad2 and Smad3, was also detected by immunohistochemical staining. Compared to the level in PD rats, TNF-α and TGF-β1 expression in the peritoneum was reduced following treatment with a high dose of *Astragalus* (Figure 5 and Table 1). The results of phospho-SMAD2/3, which are downstream of TGF-β1, confirmed the effect of *Astragalus* (Figure 6A,B) and demonstrated its inhibitory effect on the TGF-β/Smad pathway.

**Table 1:** Concentration of inflammatory cytokines in the diaphragmatic peritoneum.

Cytokines (pg per 100 μg Protein *n* = 10)	Normal Control	Peritoneal Dialysis (PD)	PD + *Astragalus*
**MCP-1**	7.9 ± 0.8	20.4 ± 1.7 *	8.7 ± 1.0 ^#^
**TNF-α**	1.4 ± 0.1	6.7 ± 0.5 *	3.3 ± 0.4 *^,#^
**TGF-β**	21.2 ± 1.8	58 ± 2.1 *	24.9 ± 1.2 ^#^

Mean ± SEM: * *p* < 0.05 compared to the normal controls; ^#^ *p* < 0.05 compared to peritoneal dialysis.

## 3. Experimental Section

### 3.1. Drugs

*Astragalus membranaceus* injections, which were made from *Astragalus mongholicus* extracts, were purchased from Chiatai Qingchunbao Pharmaceutical Co., Ltd. (Hangzhou, China) from batch No. 0508081, which contained 2 g/mL crude drug.

### 3.2. Animal Groups

Male Sprague-Dawley rats with an initial weight of 180–200 g were obtained from the Nanjing Medical University Animal Centre. All rat handing procedures were approved by the Nanjing Medical University Animal Care and Use Committee. A rat model of PD was induced by daily intraperitoneal injections with standard PD fluid (Dianeal PD-2 peritoneal dialysis solution with 4.25% dextrose, pH 5.2; Baxter Healthcare Ltd. (Singapore, Singapore) at a dose of 100 mL/kg [23]. Sixty rats were randomly divided into six groups: A group, normal control group (equal volume of saline injection, *n* = 10); B group, *Astragalus* alone group (injected intraperitoneally with 4000 mg/kg/day *Astragalus* for 7 days, *n* = 10); C group, peritoneal dialysis group (PD group, *n* = 10); D group, *Astragalus* low dose group (PD rats injected intraperitoneally with 1000 mg/kg/day for 7 days, *n* = 10); E group, *Astragalus* middle dose group (PD rats injected intraperitoneally with 2000 mg/kg/day for 7 days, *n* = 10); F group, *Astragalus* high dose group (PD rats injected intraperitoneally with 4000 mg/kg/day for 7 days, *n* = 10). *Astragalus* treatments were started on the 21st day after the first intraperitoneal injection of PD fluid, as in our previous study [25]. After 4 weeks of PD, the abdomen was opened by a midline incision, and the entire anterior abdominal wall was removed at the contralateral side to the tip of the implanted catheter. The whole tissue adjacent to the liver was fixed in 10% neutral-buffered formalin and embedded in paraffin for further examination.

### 3.3. Histology

Sections (5 μm) of the paraffin-embedded, fixed peritoneum sample tissues were deparaffinized, hydrated in ethyl alcohol, and washed in tap water. Routine HE and Masson’s trichrome staining were performed to quantify the pathological condition and peritoneum thickness [34].

### 3.4. Immunohistochemistry

The slides were deparaffinized, hydrated in ethyl alcohol, and washed in tap water. Antigen retrieval was carried out in 10 mM sodium citrate buffer for 20 min using a microwave oven. After blocking, the sections were incubated overnight at 4 °C with the primary antibodies. The two-step technique (SuperPictureTM3rd Gen IHC Detection kit; Invitrogen, CA, USA) was used for visualization, with DAB (Vector Laboratories, Burlingame, CA, USA) or AEC (Vector Laboratories, Burlingame, CA, USA) as a chromogen. Finally, the sections were counterstained with hematoxylin and mounted. The following primary antibodies were used for immunohistochemistry: anti-MCP-1 (Millipore Corporation, Billerica, MA, USA), anti-F4/80 (Macrophages/Monocytes markers in rat) (Millipore Corporation, MA, USA), anti-Fibroblast Surface Protein (FSP, Abcam, MA, USA), anti-collagen III (Abcam, MA, USA), anti-phospho-SMAD 2/3 (Abcam, MA, USA), and anti-TGF-β1 (Santa Cruz Biotechnology, Santa Cruz, CA, USA).

### 3.5. Peritoneum Thickness Measure

The thickness of the peritoneum was defined as the tissue between the mesothelial surface and the underlying muscle or parenchyma [37]. The maximal thickness of the peritoneum was measured in three Masson’s trichrome-stained tissue sections per rat, and five fields, the center of which included the area of maximal thickness, were examined under 400× magnification. The areas and perimeter lengths of the peritoneum were obtained from drawn outlines, and the average thickness was calculated from rectangular approximation based on the values for the area and perimeter in each field of view using Adobe Photoshop (Adobe Systems, TurboTax, San Diego, CA, USA) and Image-Pro Plus 5.1 software (Media Cybemetics, Silver Spring, MD, USA).

### 3.6. mRNA Expression

The tissues of the abdominal wall were homogenized under liquid nitrogen and RNA was isolated using TRIzol reagent (Life Technologies, GIBCO-BRL, Carlsbad, CA, USA). RNA was reverse transcribed into cDNA and quantitative RT-PCR was carried out as per the manufacturer’s instructions (Applied Biosystems, Foster City, CA, USA) using TaqMan Gene Expression Assays as previously described [25]. Conditions utilized for RT-PCR were as follows: 2 min at 50 °C; 10 min at 95 °C; and 40 repetitions of 15 s at 95 °C and 1 min at 60 °C. Genes measured included *MCP-1* (Mm00441242_m1), *TNF-α* (Mm00443258_m1), *TGF-β1* (Hs00998133_m1) and *18S* (Mn03928990_g1) as the reference gene. Quantification of mRNA expression of all target genes (*MCP-1*, *TNF-α*, and *TGF-β1*) was calculated using the 2^ΔΔ*C*t^ method, which employs a single calibrator sample to compare against every unknown sample’s gene expression.

### 3.7. Concentration of Inflammatory Cytokines

The tissues of the abdominal wall were homogenized in Iscove’s protein medium using a polytron and samples were centrifuged twice at 10,000× *g* at 4 °C for 15 min. The supernatants were removed and stored at 4 °C prior to the assay of MCP-1, TNF-α and TGF-β1 via ELISA the following day (R&D Systems, Minneapolis, MN, USA). The assay was performed according to the manufacturer’s instructions and all samples were run in duplicate. Total soluble protein was determined using supernatant of homogenized samples via bicinchoninic acid (BCA) protein assay (Pierce, Rockford, IL, USA). All cytokine levels are expressed as a pg per 100 μg of total protein.

### 3.8. Statistical Analysis

The results obtained from the Masson’s trichrome-stained slides were analyzed with the General Linear Model ANOVA with multiple comparisons using the Sidak adjustment procedure. The 0.05 level of probability was used as the criterion of significance. Statistical analysis was performed using SPSS (Statistical Package for the Social Sciences), version 15.0 software (IBM, Armonk, NY, USA) for Windows.

## 4. Conclusions

Our results suggest that *Astragalus* exhibits inhibitory effects on peritoneal fibrosis, and its mechanisms of action may involve both MCP-1 and the TGF-β/Smad pathway.
